# Reviewers for 2023

**DOI:** 10.4314/gmj.v58i1.2

**Published:** 2024-03

## Introduction

Reviewers of manuscripts for our journal contribute immensely to the standard and quality of the papers we publish. The Ghana Medical Journal is grateful for the voluntary service provided by the following reviewers for the year 2023.

**Table:** 

Aniekan Monday Abasiattai	Nigeria
Hanson Ababio	Ghana
Innocent Abang	Nigeria
Emem Abraham	Nigeria
Kamoru Adedokun	United States
Abiodun S Adeniran	Nigeria
Nii-Armah Adu-Aryee	Ghana
Mary Yeboah Afihene	Ghana
James Afriyie	Ghana
Jane Afriyie-Mensah	Ghana
Ezera Agwu	Uganda
Adwoa A. Agyei-Nkansah	Ghana
Francis Agyekum	Ghana
Dzifa Ahadzi	Ghana
Linda Ahenkorah-Fondjo	Ghana
Collins Stephen Ahorlu	Ghana
Olabanji Abiodun Ajose	Nigeria
Joseph Akamah	Ghana
Josephine Akpalu	Ghana
Ahmed S Ali	Saudi Arabia
Eugene Amable	Ghana
Kwadwo Amoah	Ghana
Joachim Amoako	Ghana
Mary Amoakoh-Coleman	Ghana
Isabella Amoyaw-Asamoah	Ghana
William Ankobiah	Ghana
Sampson Antwi	Ghana
Charles E. Anyanechi	Nigeria
Stephen Ajen Anzaku	Nigeria
Ivy Asantewaa Asante	Ghana
Brainard Ayisi Asare	Ghana
Marcus Inyama Asuquo	Nigeria
Foroozan Atashzadeh-Shoorideh	Iran
Yacoba Atiase	Ghana
Bismark Bright Baah	Ghana
Kenneth Kojo Baidoo	Ghana
Arjun Ballal	India
Delia Akosua Bandoh	Ghana
Antoinette A. A Bediako-Bowan	Ghana
Godness Biney	United States
Richard Biritwum	Ghana
Paa-Kwesi Blankson	Ghana
Paul Boateng	Ghana
Vincent Boima	Ghana
Joseph Bonney	Ghana
Gilbert Batieka Bonsaana	Ghana
Edmund K. K. Brakohiapa	Ghana
Benedict N. L. Calys-Tagoe	Ghana
Given Chipili	Zambia
Joe Nat A Clegg-Lamptey	Ghana
Nicola Conran	Brazil
Jonathan C. Balea Dakubo	Ghana
Joycelyn Assimeng Dame	Ghana
Rakesh Dawar	India
Steven W Day	United States
Dziedzom Komi De Souza	Ghana
Samuel Akobour Debrah	Ghana
Florence Dedey	Ghana
Leonard Derkyi-Kwarteng	Ghana
Robert Djagbletey	Ghana
Alfred Doku	Ghana
Peter Donkor	Ghana
Amoako Duah	Ghana
Fiifi Duodu	Ghana
Frank Edwin	Ghana
Ivy Ekem	Ghana
Sebastian Eliason	Ghana
Anthony Kwame Enimil	Ghana
Chinedu Ezekekwu	Nigeria
Delali Fiagbe	Ghana
Chris Opoku Fofie	Ghana
Audrey Forson	Ghana
Paa Kobina Forson	Ghana
Fred Yao Gbagbo	Ghana
Ado Danazumi Geidam	Nigeria
Anthony Godi	Ghana
Abdulaziz Hassan	Nigeria
Ibrahim Mohamed Hussaini	Nigeria
Alphonsus Rukevwe Isara	Nigeria
Ernest Kenu	Ghana
Ahmed A. Khalifa	Egypt
Star Khoza	South Africa
Emmanuel Kitcher	Ghana
Collins Kokuro	Ghana
Kwadwo Ansah Koram	Ghana
Edward Kwakyi	Ghana
Robert A Kwame-Aryee	Ghana
Margaret Lartey	Ghana
Oluwatosin Leshi	United States
Ahmed M. Maged	Egypt
Charles Mate-Kole	Ghana
Jared Mcdowall	South Africa
Samuel Mensah	Ghana
Celestine Okorome Mume	Nigeria
Hina Nafees	India
Ikenna Ndu	Nigeria
Samuel Blay Nguah	Ghana
John Nkrumah-Mills	Ghana
Portia Nkumsah-Riverson	Ghana
Kobina Nkyekyer	Ghana
Justice Nonvignon	Ghana
Josephine Nsaful	Ghana
Adetunji Obadeji	Nigeria
Kofi Obuobie	UK
Ifedayo Adeola Odeniyi	Nigeria
David Ofori-Adjei	Ghana
Edeghonghon Olayemi	Ghana
Foluwasayo E. Ologe	Nigeria
Dayo Rotimi Omotoso	Nigeria
Chalotte Osafo	Ghana
Stephen Osei-Wusu	Ghana
Edmund Ndudi Ossai	Nigeria
Isaac Kofi Owusu	Ghana
Frank Owusu-Sekyere	Ghana
Mukta Pujani	India
Peter Puplampu	Ghana
Solomon E. Quayson	Ghana
Bismark Sarfo	Ghana
Fred Stephen Sarfo	Ghana
Tarela Sarimiye	Nigeria
Benjamin Dabo Sarkodie	Ghana
Nana Ayegua H. Seneadza	Ghana
Akeem Olasunkanmi Shittu	Nigeria
Bola Lukman Solanke	Nigeria
Kenneth Tachi	Ghana
John Tampuori	Ghana
Peace Tetteh	Ghana
Mark Tettey	Ghana
Rahul VC Tiwari	India
Kwasi Torpey	Ghana
Ejiroghene Martha Umuerri	Nigeria
Jennifer E. Welbeck	Ghana
Caitlin Wiafe	Ghana

